# The Relationship Between Childhood Abuse, Personality, and Depression: A Narrative Review

**DOI:** 10.31083/AP45405

**Published:** 2025-07-30

**Authors:** Wei Luo, Bao-Liang Zhong

**Affiliations:** ^1^Department of Psychology, Wuhan University, 430072 Wuhan, Hubei, China; ^2^Department of Psychiatry, Wuhan Mental Health Center, 430012 Wuhan, Hubei, China

**Keywords:** child abuse, depression, personality, review

## Abstract

Depression is a common mental health problem that imposes a significant burden on both individuals and society. Numerous studies have shown that childhood abuse has a long-lasting detrimental effect on mental health, including the development of depression. This study reviews recent studies on the association between childhood abuse and depression, highlighting the robust causal link between childhood abuse and subsequent depression. The strength of this association varies depending on the type of abuse, with emotional abuse showing a particularly strong connection to depression. In addition, this narrative review examines the role of personality in the relationship between childhood abuse and depression. Evidence indicates that personality traits act as both mediators and moderators in this association, with neuroticism being particularly influential. Although available research evidence primarily consists of cross-sectional studies, which cannot determine the temporal sequence of childhood abuse, personality problems, and depression, the interrelations of these three variables provide a more comprehensive understanding of the etiology of depression. These insights lay the foundation for future longitudinal research and inform the development of more targeted preventive and therapeutic interventions in clinical psychiatry.

## Main Points

1. Childhood abuse, across its diverse subtypes, demonstrates significant associations with the onset of depression in adulthood, with emotional abuse showing a particularly strong connection.

2. Individuals who have experienced childhood abuse are consistently found to have a heightened predisposition toward the neurotic personality trait.

3. Personality factors were identified as exerting both mediating and moderating effects on the link between childhood abuse and depression.

## 1. Background

Depression is a prevalent mental health problem affecting the general population, including children and adolescents [1, 2]. More than 300 million people worldwide suffer from depressive disorders, accounting for 4.4% of the global population [3]. Depression causes varying degrees of harm on an individual’s cognitive, emotional, and behavioral well-being, impacting both physical and mental health and resulting in decreased social functioning or even impairment [4]. Severe depression can provoke suicidal ideation and actions, significantly distressing both the individuals and their loved ones [5]. According to the Global Burden of Diseases Study (GBD) 2019, depressive disorders are one of the leading causes of global burden, constituting the primary cause of disability-adjusted life years (DALYs) among mental disorders [6].

Numerous studies have shown that negative life events can lead to the onset of depression [7, 8, 9]. Childhood abuse is a significant early-life negative event, and extensive research has confirmed a close association between childhood abuse and adult depression. Childhood abuse refers to actions by individuals responsible for nurturing, supervising, or exercising control over children that cause actual or potential harm to the child’s health, survival, development, or dignity, including various forms of physical abuse, emotional abuse, sexual abuse, neglect, and economic exploitation [10]. Childhood abuse may increase an individual’s risk of developing depression and suicide associated with depression [11], as well as negatively impact the prognosis of depressed individuals. However, not all individuals who experience childhood abuse will develop depression or other adverse mental health outcomes [12]. The outcomes of childhood abuse depend on a combination of factors, including genetics, development, psychology, cognition, neurobiology, and environment. Among these factors, personality is crucial [13].

Personality is the integrated form of an individual’s emotions, thoughts, and behaviors [14]. Research generally holds that an individual’s personality changes to varying degrees due to the dual influence of innate genetic factors and acquired environmental factors [15, 16]. Previous studies have confirmed that childhood abuse leads to deviations from normal personality trajectories, such as higher levels of neuroticism [17, 18]. On the other hand, personality is a stable and unique personal trait affecting an individual’s response to external stimuli [19]. For example, individuals with inherently higher neurotic tendencies may react more strongly to external stimuli, have poorer emotional stability [20], and be more susceptible to the negative effects of abuse and neglect. Many researchers have examined the associations between childhood abuse, personality, and depression using diverse approaches and theoretical frameworks. This article reviews recent progress in this area.

## 2. Studies on Relationships Between Childhood Abuse, Personality, and Depression

### 2.1 Childhood Abuse and Depression

The relationship between childhood abuse and depression has been extensively reported in the international literature. Childhood abuse disrupts the normal developmental process of children [21], potentially causing lasting negative impacts throughout their lives. A meta-analysis of childhood abuse and depression revealed that 62% of the patients with major depressive disorder have a history of childhood abuse [22]. Depressed patients who have experienced childhood abuse tend to have an earlier age of onset, more severe symptoms, and a higher risk of suicide compared with those without such experiences [22].

Different types of childhood abuse exhibit varying relationships with depression. A meta-analysis published in 2023 found a significant association between the extent of childhood abuse and the severity of depressive symptoms, with a correlation coefficient (r) of 0.17. The correlation coefficient for depressive symptoms associated with specific subtypes of childhood abuse was as follows: sexual abuse (r = 0.18, *p* < 0.001), emotional abuse (r = 0.17, *p* < 0.001), physical abuse (r = 0.13, *p* < 0.001), and neglect (r = 0.08, *p* < 0.001) [23]. These findings are similar to another meta-analysis published in 2019 on childhood abuse and depressive disorders. This study highlighted the strongest association between emotional abuse and depression (odds ratio (OR) = 2.35, *p* < 0.01), followed by sexual abuse (OR = 2.11, *p* < 0.01), physical abuse (OR = 1.78, *p* < 0.01), and neglect as the least significant factor (OR = 1.65, *p* < 0.01) [24]. The results consistently indicate that each type of childhood abuse is closely associated with adult depression, with childhood sexual abuse and emotional abuse exerting a more pronounced effect. When considering prevention and intervention measures for depression, these two subtypes of abuse should be prioritized as intervention targets. In the literature, research on the associations between depression and emotional abuse or neglect is less extensive and more recent compared to studies on depression’s associations with sexual and physical abuse [25]. The inadequate research on emotional abuse and neglect may be due to the challenges in defining emotional abuse and neglect compared with other abuse subtypes [26]. Therefore, more research is needed to clarify the relationship between emotional abuse, neglect, and adult mental health outcomes.

Research on childhood sexual abuse has produced numerous findings. Previous studies have consistently demonstrated a significant association between childhood sexual abuse and adult depression [27, 28]. For example, a qualitative study by Manukrishnan and colleagues on the long-term consequences of childhood sexual abuse revealed that such abuse has enduring negative impacts on survivors, including depression, anxiety, substance abuse, and dysfunctional interpersonal relationships [27]. Zheng *et al*. [28] found that depressed female college students who experienced sexual abuse in childhood exhibited significantly more severe depressive symptoms than those who did not.

Studies on childhood physical abuse have also been conducted in the past. Chinese researchers found that college students who were physically abused in childhood exhibit higher levels of depression and are more likely to adopt negative coping strategies compared with those who were not abused [29]. A study by Lamela and Figueiredo [30] revealed that physical abuse is associated with various adult psychopathologies, including depression, anxiety, and psychoticism. However, limited studies have focused on the link between childhood physical neglect and depression. Existing research often incorporates physical neglect within the broader framework of childhood abuse or considers it as part of physical abuse. Drawing from available research and theories on childhood abuse, it is hypothesized that experiences of childhood neglect are associated with later depression [31, 32].

Emotional abuse during childhood has received research attention only in recent years. Some studies consolidate emotional abuse and neglect into one category for examination, while others label it as psychological abuse and neglect, delineating forms of childhood abuse that do not involve physical or sexual contact [33, 34]. Regardless of terminology, emotional or psychological abuse is linked to subsequent depression. Ye *et al*.’s study [35] among adolescents revealed that childhood emotional abuse and neglect directly predicts depression or indirectly predicts it through avoidant attachment, self-blame, and blaming others. Christ *et al*.’s study [36] highlighted that emotional abuse is associated with affective dysregulation and depressive symptoms in female college students, with emotional abuse indirectly influencing depression through affective dysregulation and interpersonal problems.

### 2.2 Childhood Abuse and Personality

Personality is a distinct psychosomatic construct developed during the process of socialization, embodying an individual’s inherent behavioral tendencies and encompassing the full spectrum of psychological characteristics [14, 36]. The construction of personality is intricately intertwined with genetic predispositions as well as environmental factors.

Some scholars highlight that childhood abuse not only affects an individual’s personality by modifying neurobiological elements such as neurons and synapses [37], but also profoundly influences personality through alterations in cognitive schemas, attachment styles, levels of socialization, and emotional states [38]. Previous research has shown that childhood abuse is associated with the development of certain personality disorders in adulthood, e.g., borderline personality disorder [39] and antisocial personality disorder [40]. Even if the impairment in personality caused by childhood abuse is not severe enough to meet the diagnostic criteria for a personality disorder, childhood abuse may produce varying degrees of deviations from normal personality traits in the person experiencing it, as supported by the results of the Big Five Personality Questionnaire. Most studies consistently show that individuals with a history of childhood abuse are associated with higher levels of neuroticism, with some studies showing an association with lower levels of extraversion, while the findings for openness, conscientiousness, and agreeableness are not consistent.

Children subjected to prolonged exposure to traumatic environments, characterized by persistent physical and psychological threats, are more prone to unstable emotions, leading to heightened neurotic tendencies in their personality. Most studies suggest that the severity of childhood abuse experienced is positively correlated with the intensity of an individual’s neurotic tendencies [41, 42]. Research on the correlation between childhood abuse experiences and personality traits among college students revealed that those with higher abuse scores exhibited elevated levels of psychoticism and neuroticism compared with those with lower abuse scores [43]. Additionally, a positive relationship was observed between physical abuse, physical neglect, emotional abuse, emotional neglect, and both neuroticism and psychoticism [43]. Another study among first-degree relatives of patients with major depressive disorder also demonstrated positive links between both neuroticism and psychoticism and total childhood abuse, as well as emotional neglect, physical neglect, and sexual abuse [44].

Persistent abuse and neglect from parents may destroy one’s secure attachment, lead children to internalize blame, forming beliefs that their mistreatment was a reflection of their own inadequacy, lowering their self-assessment and self-esteem [45, 46], hindering self-acceptance and expression, and potentially fostering introversion. Yang and colleagues [47] used both quantitative and qualitative approaches and ascertained a significant connection between childhood abuse and low extraversion. Zhang *et al*. [48] revealed that childhood abuse is negatively related with extraversion.

The correlation between childhood abuse and adult antisocial personality disorder implies a potential link between lower levels of agreeableness and childhood abuse. Individuals with a history of childhood abuse, potentially due to exposure to traumatic upbringing, may mirror their parents’ behavioral patterns, resulting in personalities characterized by reduced empathy, genuineness, and agreeableness. However, research findings regarding the Big Five personality traits have not reached a consensus. Alnassar *et al*.’s study [42] showed that emotional and sexual abuse significantly contribute to agreeableness, while emotional neglect negatively contribute to agreeableness. Zhu *et al*. [49] found that agreeableness is negatively correlated with childhood abuse. Another study in patients with depression revealed that agreeableness is negatively correlated with emotional abuse, but not associated with sexual abuse, physical abuse, or neglect [50].

Openness, a personality trait closely linked to cognitive processes, is associated with cognitive flexibility, complexity, emotional sensitivity, and emotional intelligence, as per research by McCrae and Sutin [51, 52]. Childhood abuse could engender negative cognitive patterns, such as dysfunctional attitudes, leading individuals to interpret external information in a distorted and pessimistic manner. This fosters automatic and inflexible thinking, thus limiting openness to the external world. The study by Xu [53] indicates significant discrepancies in agreeableness and openness among high school students with varying trauma histories, with increased trauma being associated with lower scores in openness. However, a study suggests that openness is not associated with childhood abuse [54].

Alnassar *et al*. [42] examined conscientiousness, another personality trait potentially linked to childhood abuse, and found that individuals with a history of childhood emotional neglect tend to score lower in conscientiousness. Zhu *et al*.’s study [49] demonstrated that various types of childhood abuse are negatively correlated with conscientiousness. Similar to openness and agreeableness, research indicates that only emotional abuse is negatively associated with conscientiousness, while physical and sexual abuse showed no significant relationship with this personality trait [54]. In summary, a limited number of studies have identified a correlation between childhood abuse and conscientiousness, but most do not explain the mechanisms underlying these associations. Therefore, further research is needed to investigate and clarify these complex relationships.

### 2.3 Personality and Depression

An individual’s health and behaviors are closely linked to personality [55]. Prior research has demonstrated a significant association between personality traits and depression. Fausor and colleagues used the Revised NEO Personality Inventory and the Beck Depression Inventory to examine the relationship between personality and depression. Their study showed a positive correlation between depression and neuroticism, and negative correlations with extraversion, with no significant correlations with openness, agreeableness, and conscientiousness [56]. These results are similar to the findings of Lee and Song [54], except for the low but significant negative correlation they identified between conscientiousness and depression. Du and colleagues [57], in a survey among university students in Guangdong province, China, found that depression was significantly and positively correlated with neuroticism and negatively correlated with extraversion, openness, and conscientiousness. Agreeableness did not show a significant correlation with depression [57]. While previous studies have consistently shown a substantial link between neuroticism, extraversion, and depression, there is less consensus regarding conscientiousness, openness, and agreeableness.

Neurotic individuals demonstrate heightened reactivity to external stimuli, experience pronounced emotional fluctuations [58], and are susceptible to anxiety. Higher neuroticism scores predispose individuals to intensified reactions to external stressors, increasing susceptibility to depressive symptoms. In contrast, extraversion acts as a protective factor against depression. Extroverted individuals typically exhibit positive emotions, handle interpersonal relationships adeptly [59], and thereby access more support in times of need, which can mitigate the severity of depressive symptoms [60].

Conscientiousness is associated with impulse control and regulation. Low conscientiousness, due to a lack of willpower and achievement, can lead to feelings of frustration and depression [58, 61]. However, conscientiousness is also linked to perfectionism [62], which is a risk factor for depression [63]. Agreeableness, characterized by empathy and helpfulness [64], contributes to a harmonious interpersonal relationships on daily life, potentially reducing the incidence or severity of depression [65]. On the other hand, high levels of agreeableness may impede problem-solving skills, impacting mental health levels. Openness reflects cognitive traits, with individuals high in this trait engaging more with the external world, potentially enhancing their resilience to cope with stress [66], and reducing the risk of depressive symptoms by resolving internal conflicts and managing stress more effectively. However, there are also a few studies showing a positive association between openness and depression [67, 68].

## 3. The Dual Roles of Personality in the Relationship Between Childhood Abuse and Depression

The diathesis-stress model of depression posits that depressive episodes are influenced by the combined effects of stressors and predisposing factors (diathesis) [69]. Personality is a psychosomatic organization characterized by both stability and uniqueness [70], and a significant diathesis factor that affects an individual’s response to external stimuli [19], leading to different perceptions and coping mechanisms when facing similar situations.

Several studies have explored the role of personality as a mediating factor in the relationship between childhood abuse and depression. For example, Hayashi and colleagues [50], in their study of 113 patients with major depression, found that childhood abuse indirectly predicts depressive symptoms through the mediating effects of personality traits such as neuroticism, extraversion, and conscientiousness. In 2024, Qin and colleagues [71] conducted a survey of 1272 college students to examine the impact of childhood abuse and neuroticism on depressive symptoms. The results showed that childhood abuse could directly affect depressive symptoms and indirectly influence the development of depressive symptoms through neuroticism. Zhang and colleagues [48] confirmed the existence of the “childhood trauma-personality traits-depressive symptoms” pathway through their research on Chinese adolescents, with neuroticism and extraversion mediating the relationship between childhood trauma and depressive symptoms.

Although the formation of personality is influenced by individual life experiences and social practices, it is also closely related to an individual’s biological and genetic factors, with variations in postnatal physical and mental health levels due to inherent differences in personality traits. Some studies have also examined personality as a moderating factor in the relationship between childhood abuse and depression [72, 73, 74].

In 2020, Hu *et al*. [72] found that neuroticism moderated the relationship between childhood abuse experiences and depression among college students, with those high in neuroticism being more prone to depressive moods after experiencing childhood abuse compared with those with lower levels of neuroticism. However, this study did not measure other personality types and therefore had no data on their moderating effects. In 2022, Chu *et al*. [73] examined the moderating role of neuroticism in the relationship between childhood trauma and current depression among college students and confirmed its significant moderating effect. However, this study combined various types of childhood abuse into a single index, failing to clearly demonstrate whether the moderating effect of neuroticism varies according to the subtype of childhood abuse. Other research has examined the moderating role of neuroticism in the relationship between strict maternal punishment and depressive symptoms among nursing students, showing that higher levels of neuroticism lead to a more significant moderating effect [74]. Overall, only a small number of studies have addressed the moderating role of personality in the relationship between childhood abuse and adult depressive symptoms, primarily focusing on the dimension of neurotic personality, with very few studies examining the moderating effects of personality in the relationships between various types of childhood abuse and depression.

It is clear that both childhood abuse and personality traits can contribute to adult depression. In addition to the interrelationships among these three factors, it is imperative to consider that their relationships may be influenced by a host of closely intertwined factors. One particularly salient factor is the gender-specific difference. For example, studies have generally revealed that females are more prone to experiencing sexual abuse than males [75, 76]. Variations in the intensity and recurrence of exposure to childhood abuse can result in divergent health consequences between males and females. Previous studies have also shown that certain personality-related traits inherently exhibit gender differences and tendencies, leading to varying susceptibilities to depression [77, 78]. For instance, traits related to emotion (e.g., alexithymia, expression suppression) are implicated. Li *et al*. [77] explored the relationship between parental bonding, personality traits (alexithymia), and mental health, and found that poor paternal bonding can more directly contribute to mental health problems compared with maternal bonding. It can also indirectly affect mental health by influencing the development of alexithymia, particularly in males [77]. Cognitive-related personality factors also exhibit gender differences, such as attributional styles. Other research has shown that women are more likely to engage in internal attributions and self-blame when faced with childhood abuse [79]. Consequently, it is crucial to account for the gender disparities between the individuals involved in child maltreatment, recognizing that these differences can lead to distinct responses to abuse. The interactive effects of gender may elicit varied reactions to child maltreatment, which in turn contribute to a spectrum of outcomes. In addition to gender, factors such as age and socioeconomic status should also be considered for future study.

Considering the important implications of personality traits in the relationship between childhood abuse and adult depression, interventions targeting the personality traits of individuals with a history of childhood abuse may effectively reduce the risk of depression. In fact, several typical psychotherapeutic interventions have been shown to contribute to the healthy development of personality, to varying degrees. A meta-analysis confirmed that both pharmacological and psychosocial interventions can promote changes in personality traits, particularly in neuroticism and extraversion [80]. Some interventions may have their unique advantages in dealing with individuals with a history of childhood abuse. A study has demonstrated that the combination of Cognitive Behavioral Therapy with Art Psychotherapy can facilitate the expression of traumatic feelings in women who have experienced childhood sexual abuse, reducing psychological stress, which in turn helps to enhance emotional stability (neuroticism) [81]. Mindfulness therapy has been shown to reduce rumination and distress in individuals with a history of childhood abuse, decrease self-blame and shame, and boost self-esteem, all of which may be related to increased extraversion [82].

One common methodological limitation in existing studies is that most studies are cross-sectional surveys, which introduces the potential for recall bias in the information collected about childhood abuse exposure. Second, many studies have not adequately controlled for confounding factors, thereby potentially introducing confounding bias into the relationship between childhood abuse and adult depression. Third, due to the lack of longitudinal studies, there is insufficient evidence supporting the temporal sequence of childhood abuse leading to personality deviations, which in turn may trigger depression through mediating mechanisms. Longitudinal cohort studies are needed to clarify the temporal sequence between childhood abuse, personality deviations, and depression. Fourth, the cumulative exposure duration and age of exposure to childhood abuse may also be key factors influencing adult depression, yet current research seldom reports on the relationship between these two factors and adult depression. Fifth, it is not uncommon for children to experience two or more types of childhood abuse. However, the interaction or synergistic effects of different types of abuse and their relationships with adult depression are rarely examined in the literature.

Future studies should adopt a large-sample longitudinal cohort methodology that follows children from childhood into adulthood. These studies should conduct detailed assessments of abuse, including type, duration, and age of exposure, as well as personality traits and their developmental trajectories. In addition, they should examine the onset of depression and account for confounding factors to fully elucidate the mechanisms through which childhood abuse impacts adult depression and the mediating or moderating role of personality. Research in this area could provide a theoretical basis for early intervention and preventive measures for adult depression and offer insights for optimizing psychological treatment plans in clinical psychiatry.

## 4. Conclusion

Currently available studies generally demonstrated associations between childhood abuse, depression, and personality traits. A substantial body of work has identified childhood abuse as a significant risk factor for the onset of depression, with the strength of this association varying across the type of abuse. Meta-analytic evidence particularly highlights the predictive power of emotional and sexual abuse on depression. Childhood abuse is also associated with a variety of personality traits, with a heightened tendency towards neuroticism frequently observed in individuals with a history of abuse. Additionally, the interplay between personality and the impact of childhood abuse on depression has been explored extensively, suggesting that personality not only mediates but also moderates this relationship. This indicates that both the stability and variability of personality traits significantly influence the interaction between childhood abuse and the risk of depression. However, further empirical research is needed to deeply examine the specific roles and strengths of different personality types.
